# Changes in patellar tendon complaints and shear wave velocity patterns among competitive alpine skiers during a 4-year post-growth spurt follow-up

**DOI:** 10.3389/fphys.2024.1401632

**Published:** 2024-07-08

**Authors:** Jonas Hanimann, Daniel P. Fitze, Tobias Götschi, Stefan Fröhlich, Walter O. Frey, Eling D. de Bruin, Reto Sutter, Jörg Spörri

**Affiliations:** ^1^ Sports Medical Research Group, Department of Orthopaedics, Balgrist University Hospital, University of Zurich, Zurich, Switzerland; ^2^ University Centre for Prevention and Sports Medicine, Balgrist University Hospital, University of Zurich, Zurich, Switzerland; ^3^ Department of Health Sciences and Technology, ETH Zürich, Zurich, Switzerland; ^4^ Orthopaedic Biomechanics Laboratory, Department of Orthopaedics, Balgrist University Hospital, University of Zurich, Zurich, Switzerland; ^5^ Institute for Biomechanics, ETH Zurich, Zurich, Switzerland; ^6^ Department of Health, OST—Eastern Swiss University of Applied Sciences, Zurich, Switzerland; ^7^ Department of Radiology, Balgrist University Hospital, University of Zurich, Zurich, Switzerland

**Keywords:** shear wave elastography, tendinopathy, overuse injury, athlete, alpine skiing

## Abstract

Patellar tendon (PT) complaints are frequent in competitive alpine skiers and such complaints are characterized by a long-lasting affection. Since PTs are subject to maturation up to 1–2 years after growth spurt, this early career stage may be decisive for the further course of complaints. The aim of this study was to investigate the evolution of PT complaints and shear wave velocity patterns among competitive alpine skiers during a 4-year post-growth spurt follow-up. The PT complaints and SWV patterns of forty-seven skiers were analysed at baseline (i.e., immediately after their peak height growth at 13–15 years of age) and were re-analysed at 4-year follow-up. The PTs were scanned via three-dimensional SWE. Symptomatic skiers were identified based on pain sensation under loading and pressure-induced pain around the PT. The prevalence of PT complaints decreased from 29.8% at baseline to 12.8% at follow-up (Pearson’s χ^2^ = 9.429; *p* = 0.002). SWV decreased from the baseline assessment to the follow-up in the proximal and distal regions (*p* < 0.05). SWV coefficient of variation (CV) in the distal and mid-portion regions was greater at baseline than at follow-up (*p* < 0.05). At the follow-up assessment, compared to “healthy” skiers, “healed” skiers who recovered from PT complaints had lower SWVs in the proximal region (*p* = 0.020) and greater SWV CVs in the proximal region (*p* = 0.028). Moreover, symptomatic skiers had significantly greater SWV CVs in the mid-portion region than did “healthy” subjects with no history of PT complaints (*p* = 0.020). The average SWV was negatively correlated with the SWV (proximal: *r* = −0.74, *p* < 0.001; mid-portion: *r* = −0.37 *p* = 0.011; and distal: *r* = −0.58, *p* < 0.001). The occurrence of PT complaints decreased over a 4-year post-growth spurt follow-up. “Healed” skiers who were symptomatic at baseline had an even greater average decrease in the proximal and mid-portion SWV than “healthy” skiers with no history of PT complaints. This may lead to the hypothesis that PT complaints in adolescent skiers are not self-eliminating towards the end of adolescence, as at least structural irregularities appear to persist for several years after the onset of initial symptoms. Furthermore, “healed” and symptomatic tendons exhibited increased SWV variability, supporting the hypothesis that SWV CV may provide additional valuable information on the mechanical properties of PTs affected by overuse-related complaints.

## 1 Introduction

Tendons connect muscle to bone and, thus, are responsible for force transmission and are crucial in human movement and locomotion. Moreover, through energy storage and release cycles, tendons enhance muscle function and contribute to movement efficiency (Roberts, 2002). Since tendon pathologies are located on a partly reversible continuum, both physiological and pathological processes are of great interest (Cook and Purdam, 2009). The exertion of tensile stress on tendons increases both collagen synthesis and degradation rates, and when applied repetitively, can promote tendon remodelling and thickening (Magnusson et al., 2010; Bohm et al., 2015). Moreover, the structural integrity of the tissue increases through enhanced enzymatic cross-linking (Svensson et al., 2016; Passini et al., 2021). However, repetitive and excessive overloading can lead to the accumulation of tendon damage and, in combination with inadequate repair mechanisms, can trigger degenerative pathological processes (Cook and Purdam, 2009). More precisely, these processes include neovascularization, tenocyte rounding and proliferation, an increase in collagen type III fibres, and fibrocartilaginous changes through the decomposition of glycosaminoglycans (Maffulli et al., 2000; Fredberg and Stengaard-Pedersen, 2008; Attia et al., 2014; Snedeker and Foolen, 2017). Compared to collagen type I fibres, collagen type III fibres exhibit a decreased ability to form cross-links and, therefore, impair fibre orientation and mechanical strength within the tendon (Maffulli et al., 2000). Highly hydrophilic side chains within glycosaminoglycans increase the water content within the tendon (de Mos et al., 2007). Eventually, these degenerative processes impair the mechanical properties of tendons (Ooi et al., 2016; Finnamore et al., 2019) and thus favour pathologic tendons for traumatic injuries (Cook and Purdam, 2009).

Despite promising results with rehabilitation regimens in patellar tendinopathy and the known ability of tendons to adapt to different stimuli, it remains unclear whether tendons can heal at a structural level in advanced tendinopathy (Cook and Purdam, 2009; Cook et al., 2016). The current proposal is that tendon repair occurs to a certain degree but not fully, and rather in the early stages of degeneration (Rudavsky and Cook, 2014; Cook et al., 2016). Moreover, it is assumed that degenerative tendon portions are mechanically silent and not involved in remodelling; consequently, other portions are subject to stronger stimuli and consequently adapt to and compensate for the degenerated portions (Thornton and Hart, 2011; Rudavsky and Cook, 2014; Cook et al., 2016). Tendon assessments by shear wave elastography revealed that shear wave velocity (SWV), a measure that is associated with tendon stiffness (the higher SWV the stiffer the tissue), is decreased in subjects suffering from patellar tendinopathy (Dirrichs et al., 2016; Dirrichs et al., 2018; Götschi et al., 2023). Additionally, the aforementioned processes theoretically lead to an increase in the variability of the shear wave velocity (SWV CV) within the tendon; however, development over time of this phenomenon has not yet been shown.

The patellar tendon (PT) is among the most common tendons affected by clinical complaints (Florit et al., 2019), and adolescent athletes and competitive alpine skiers in particular are at high risk (Simpson et al., 2016; Fröhlich et al., 2020). Because the term “tendinopathy” was previously defined as persistent proximal PT pain associated with mechanical loading independent of structural changes/imaging abnormalities (Scott et al., 2020) and because PT tendon complaints in adolescent athletes may affect anatomical regions other than the proximal region (Cassel et al., 2015), we use the term “PT complaints” hereinafter for symptoms of motion- and palpation-induced tenderness in either the proximal, mid-portion or distal region of the PT. In a cross-sectional study of competitive alpine skiers aged 13–15 years, 47.2% of all skiers had suffered from at least one overuse-related knee complaint in the last 12 months (Fröhlich et al., 2020). Approximately one-third of these complaints were distal PT complaints, one-third were proximal PT complaints, and one-third were unspecified overuse complaints (Fröhlich et al., 2020). In a similar cohort, skiers suffering from proximal and distal PT complaints showed decreased regional shear wave velocity (SWV) in the affected regions, and subjects with distal complaints additionally had lower SWV in the proximal region (Götschi et al., 2021). Because skiers who suffer from such complaints can be severely restricted in their sports performance development and miss out on training and competition opportunities in performance-relevant phases, the importance of primary prevention of PT complaints at the youth level is obvious.

How such PT complaints in skiers further progress throughout and towards the end of adolescence is, however, not fully understood. While it can be assumed that especially growth-related PT complaints such as Osgood-Schlatter disease and associated pain symptoms decrease towards the end of adolescence, their mid- and long-term development (especially on a structural level) remains poorly understood. In a similar context but in heathy subjects, previous literature has reported that proximal and distal PT portions mature particularly 1–2 years after growth spurt (Ducher et al., 2010a; Ducher et al., 2010b; Rudavsky et al., 2017), and that the formation of the tendon core occurs primarily during height growth, while it becomes relatively inert after adolescence (Heinemeier et al., 2013). Regardless of this, however, tendons are likely to retain their basic ability to adapt to exercise-induced stresses (Maganaris et al., 2017; Quinlan et al., 2019). This implies that the youth stage in a high-performance sports career may be decisive for the subsequent course of PT complaints and, in this regard, SWE may help to assess the biomechanical properties of PTs and may provide further insights into the pathophysiology and the progression of such complaints. However, it remains to be investigated the PTs of asymptomatic and symptomatic competitive alpine skiers develop in terms of their clinical manifestations and mechanical proprieties beyond the growth spurt, i.e., when skiers are aged 17–19 years, which is largely unknown at present.

Accordingly, in the present study, we reexamined a subsample of the cohort on which the abovementioned studies (Fröhlich et al., 2020; Götschi et al., 2023) were based as part of a 4-year prospective follow-up study after the growth spurt using the same methods. The specific aims of this study were 1) to describe the prevalence and 4-year progression of PT complaints from baseline to the 4-year follow-up assessment in adolescent competitive alpine skiers; 2) to investigate the 4-year changes and potential sex differences in PT SWV in asymptomatic skiers according to the tendon region; 3) to analyse the differences in average SWV and SWV variability patterns between skiers who were symptomatic at baseline but who were healed at the 4-year follow-up and those with no history of patellar tendon complaints; and 4) to assess potential associations between SWV average and SWV variability.

## 2 Materials and methods

### 2.1 Participants and study design

Four years after the baseline assessment, forty-seven participants (22 females, 25 males) from the original cross-sectional study sample of 108 competitive alpine skiers examined in previous studies (Fröhlich et al., 2020; Götschi et al., 2023) participated in a follow-up assessment. At the baseline assessment, the subjects were 14.8 ± 0.6 years old. Both, the baseline and follow-up assessments were performed at the beginning of the competition season. While the entire baseline cohort was invited to participate in the 4-year follow-up assessment, 44 skiers were not interested in remeasurement, and the data of 17 skiers were discarded due to incomplete data or logistical issues, resulting in a total prospective sample size of 47 skiers aged 17–19 years at the follow-up assessment (aged 13–15 years at the baseline assessment). No participants were excluded due to the official exclusion criteria of this study being diagnosed with acute knee sprain at the time of the assessments or systemic pathologies such as inflammatory arthritis and diabetes mellitus due to their influence on joint-related health problems and/or tendon characteristics. This study was approved by the cantonal ethics committee Zurich (KEK-ZH-NR: 2017-01395). All participants were informed about the measurement procedures and provided written consent. If participants were below the age of 14 years, the participants’ legal guardians/next of kin provided written informed consent for them.

### 2.2 Clinical examinations

Symptomatic and asymptomatic skiers were identified by clinical examination based on two criteria, namely, pain sensation in the preceding year under loading (retrospective question on self-perceived pain sensation [yes; no] at baseline and follow-up) and acute palpation-induced pain around the PT at the baseline and follow-up examination days, whereas based on the latter, the affected PT region was recorded. Palpation-induced pain around the PT [yes; no] was assessed at baseline and follow-up by different assessors but based on the instructions and after training by the same experienced sports physician. Subjects were asked whether they felt pain on palpation of the proximal, central and distal aspects of the PT. Additionally, participants’ height (cm) and body mass (kg) were assessed by means of a measuring tape and a scale, respectively. The participants’ date of birth was known from the baseline assessment, and the age at follow-up assessment day was calculated for each participant. In the following sections, “healed” subjects are defined as skiers who were symptomatic at the baseline assessment but asymptomatic at follow-up, “symptomatic” subjects as skiers who were symptomatic at the follow-up assessment, and “healthy” subjects as skiers who were asymptomatic at both the baseline and follow-up assessments.

### 2.3 Ultrasound assessment

The PT SWV of all participants was assessed via US-based three-dimensional (3D) shear wave elastography (SWE). In a proof-of-concept study with ten healthy subjects, 3D SWE showed good inter-operator reliability (ICC: 0.736) and very good inter-day reliability (ICC: 0.904) in PT assessments (Götschi et al., 2021). In the same study, the measurement uncertainties of this method were reported to be 0.303 m*s^−1^ and 0.440 m*s^−1^ for inter-day and inter-operator assessments, respectively (Götschi et al., 2021). The assessments were performed by a single experienced examiner, and both knees of all participants were assessed. The assessment and data processing procedures followed the protocol of our proof-of-concept study (Götschi et al., 2021) and the baseline studies (Fröhlich et al., 2020; Götschi et al., 2023). In brief, participants were in the supine position with their knees bent approximately 20° to ensure a comfortable and relaxed position, and adjustable support was placed below the knees. An US device capable of high frame-rate US acquisition was used (Aixplorer^®^ Ultimate, Supersonic Imagine, Aix-en-Provence, France) combined with a linear transducer (SL18-5). The US transducer was aligned with the tendon fibre axis and held perpendicular to the skin. Optimal transducer positioning in terms of alignment with the tendon fibres was supported by maximizing the fibrillar appearance of the tendon tissue. Using an optical tracking system (FusionTrack 500, Atracsys LLC), the position and orientation of the US transducer, which was equipped with a customized shell with four optical markers, were recorded simultaneously with image acquisition. 3D data representation was based on a voxelised grid filled with two-dimensional data (SWE and US brightness-mode (B-mode)) using a weighted averaging scheme (MATLAB 2020a; The MathWorks, Inc., Natick, MA, United States).

Segmentation of the tendon was performed based on the B-mode volume (Götschi et al., 2023). Regional tendon analysis was enabled by dividing the masked SWE reconstruction into two regions (proximal, mid-portion, and distal), with cuts shifted 10 mm toward the tendon center from the proximal and distal insertion (Götschi et al., 2021). The proximal insertion was defined as the most distal attachment point of the patella to the PT, the distal insertion was defined as the most proximal attachment of the tibia to the PT, and the respective landmarks were set manually during segmentation. Weighted mean values for the respective regions (proximal, mid-portion, and distal) were used for statistical analysis. Moreover, as a measure of SWV variability in the corresponding tendon regions, the coefficient of variation (CV) was calculated and compared.

### 2.4 Statistical analysis

All the statistical analyses were performed with IBM SPSS software version 28 (IBM, Armonk, United States), and the figures were created with R (version: 2023.09.1 + 494). The Shapiro‒Wilk test was used to test the assumption of normality. For “healthy” skiers, mean values between the left and right limbs were used. For symptomatic skiers and “healed” skiers who were symptomatic at baseline but asymptomatic at follow-up, the values from the affected tendon were used, and the contralateral side was not used for further analysis.

The prevalence of skiers suffering from PT complaints was expressed as the absolute number and percentage of affected individuals. Prevalence differences between the baseline assessment and the 4-year follow-up were tested by Pearson χ^2^ tests (*p* < 0.05) for proximal, distal, and any patellar tendon complaints. Absolute differences were calculated as Difference=Follow−Up−Baseline ; thus, positive values represent an increase, and negative values represent a decrease. Similarly, % differences were calculated by the formula $Difference\;\left(\%\right)=\frac{Follow-Up\;-Baseline}{\frac{Follow-Up+Baseline}{2}}*100$ . Again, positive values represent an increase, and negative values represent a decrease.

All metric data are presented as group means and standard deviations. Repeated-measures multivariate analysis of variance (MANOVA) with Bonferroni correction for pairwise comparisons was used for the analysis of potential region, timepoint and sex differences. The within-subject factors were region (proximal; mid-portion; and distal) and timepoint (baseline assessment; 4-year follow-up assessment), and the between-subject factor was sex (females vs. males). Pairwise comparisons for within-subject factors were assessed using paired sample t tests, and pairwise comparisons for between-subject factors were assessed using unpaired sample t tests. Group differences in the region-specific average SWV and CV SWV between “healed” skiers, “symptomatic” skiers, and “healthy” skiers were assessed using Kruskal-Wallis test, and Dunn-Bonferroni tests with Bonferroni-correction were performed for *post hoc* tests. The statistical significance was set at α = 0.05. Correlations between the average SWV and the CV of the SWV were assessed for the proximal, mid-portion, and distal regions; Pearson correlation was used, and according to Cohen (1992), the following criteria were applied: R = 0.50 (*strong*), R = 0.30 (*moderate*), and R = 0.10 (*small*) (Cohen, 1992).

## 3 Results

### 3.1 Prevalence and 4-year progression of PT complaints from baseline to the 4-year follow-up assessment in adolescent competitive alpine skiers

**FIGURE 1:**
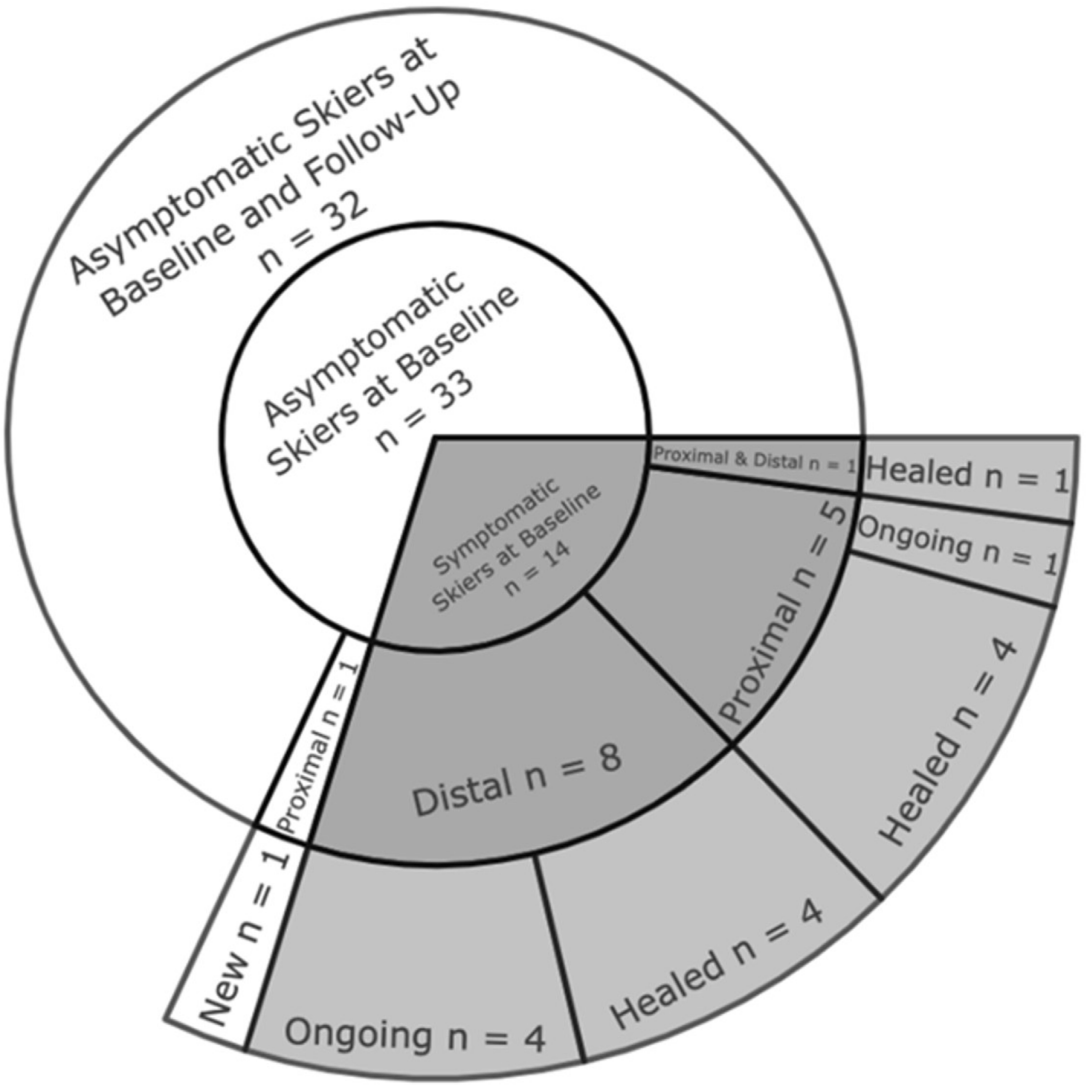
Sunburst chart presenting the progression of PT complaints in competitive alpine skiers from baseline to the 4-year follow-up assessment.

Figure 1 provides an overview of the skiers with and without PT complaints at the baseline assessment and its progression towards the 4-year follow-up assessment. Of the 47 skiers assessed at baseline, 33 skiers were asymptomatic. Fourteen skiers suffered from PT complaints, of whom 8 had distal complaints, 5 had proximal complaints, and one was affected by complaints in both the distal and proximal regions. Of the 8 skiers with distal PT complaints, 4 had healed, and 4 had ongoing complaints at the 4-year follow-up assessment. Of the 5 skiers with proximal PT complaints, 4 were healed, and one had ongoing complaints. From baseline to the 4-year follow-up assessment, there was one skier with a newly developed PT complaint in the proximal region.

Table 1 summarizes the numbers and percentages of skiers affected by PT complaints at the baseline assessment and at the 4-year follow-up assessment. With continuing adolescence, the number and percentage of skiers affected by PT complaints decreased from the baseline assessment (skiers aged 13–15 years) to the follow-up assessment (skiers aged 17–19 years) for all regions. However, Pearson χ^2^ tests revealed significant differences in the proportions of PT complaints in the distal region and in the proportions of PT complaints in any region (*p* < 0.05).

**TABLE 1:** The numbers and percentages of skiers affected by patellar tendon complaints at the baseline assessment and the 4-year follow-up assessment. One subject had both proximal and distal patellar tendon complaints and was listed in both regions.

*Region*	*4-year Follow-up (n = 47)*	*Baseline (n = 47)*	*χ* ^*2*^	*p-value*
***Proximal***	2 (4.3%)	6 (12.8%)	2.600	0.241
***Distal***	4 (8.5%)	9 (19.2%)	18.460	<0.001
***Any***	6 (12.8%)	14 (29.8%)	9.429	0.002

The data are expressed as the number of affected skiers, with percentage values in brackets. The level of significance for differences between the measurement timepoints was determined with Pearson’s χ^2^ tests.

### 3.2 Four-year changes and sex differences in the PT shear wave velocity of “healthy” skiers according to tendon region

On a multivariate level, there were significant differences in the average SWV between the regions (F (2,60) = 56.64, *p* < 0.001, η^2^ *p* = 0.654). There were also significant differences between the baseline assessment and the 4-year follow-up assessment (F (1, 30) = 10.51, *p* = 0.003, η^2^ *p* = 0.259). There was an interaction effect for the timepoint*region of the assessment (F (2, 60) = 14.84, *p* < 0.001, η^2^ *p* = 0.331), but there was no sex difference. Multivariate analysis of the region- and timepoint-dependent comparisons of the SWV CVs revealed significant differences between the proximal, mid-portion and distal regions (F (2, 60) = 77.03, *p* < 0.001, η^2^ *p* = 0.720). Moreover, SWV CV differed between the baseline assessment and the 4-year follow-up assessment (F (1, 30) = 33.91, *p* < 0.001, η^2^ *p* = 0.531). The interaction effect region* timepoint (F (2, 60) = 6.56, *p* = 0.003, η^2^ *p* = 0.179) was significant. Again, no differences between the sexes were found. All effect sizes were f > 0.4 and thus considered *strong* for all the presented analyses.

*Post hoc* pairwise comparisons are depicted in Table 2. Although there were no sex differences in height or weight at the baseline assessment, male skiers were significantly taller and heavier than female skiers at the 4-year follow-up. Moreover, for male skiers, the increase in height and weight from baseline to follow-up was greater than that for female skiers. There were no sex differences regarding the average SWV or the SWV CV in any region or at any timepoint. The average PT SWV decreased from the baseline assessment to the 4-year follow-up assessment in the proximal (*p* = 0.013, and *p* = 0.014) and distal regions (*p* = 0.005 and *p* = 0.004) in female and male skiers. The SWV CV in the distal (*p* = 0.001 and *p* = 0.003) and mid-portion (*p* < 0.001 and *p* = 0.004) regions was significantly smaller at the 4-year follow-up than at baseline in female and male subjects, respectively. In the proximal region, the SWV CV was decreased in female skiers (*p* = 0.020).

**TABLE 2:** Baseline characteristics and shear wave velocity (SWV) patterns at baseline and at the 4-year follow-up divided into subgroups of male and female skiers.

	*Baseline (n = 32)*		*4-year Follow-up (n = 32)*	
	*Female (n = 15)*	*Male (n = 17)*	*Female (n = 15)*	*Male (n = 17)*
***Age [years]***	14.79 (±0.66)	14.85 (±0.55)	19.08 (±0.59)	18.87 (±0.64)
***Height [cm]***	163.50 (±6.12)	168.41 (±6.24) #	165.67 (±5.37) *	178.59 (±6.24) *#
***Weight [kg]***	55.63 (±6.35) #	58.59 (±11.51) #	60.80 (±7.42) *#	77.06 (±8.64) *#
avg SWV_proximal [m*s ^***-1***^ ***]***	11.52 (±1.25) #	11.75 (±1.30) #	10.51 (±0.43) #	10.76 (±1.05) #
avg SWV_mid-portion [m*s ^***-1***^ ***]***	10.11 (±1.09)	9.77 (±1.50)	9.98 (±0.76)	9.82 (±1.55)
avg SWV_distal[m*s ^***-1***^ ***]***	11.55 (±1.26) #	11.57 (±1.12) #	10.25 (±0.63) #	10.28 (1.09) #
***CV_SWV_proximal [%]***	25.15 (±4.34) #	25.90 (±4.18)	22.05 (±2.21) #	24.57 (±5.07)
***CV_SWV_mid-portion [%]***	21.23 (±3.00) #	22.57 (±4.14) #	14.49 (±2.69) *#	17.10 (±4.14) *#
***CV_SWV_distal [%]***	23.76 (±3.41) #	25.32 (±4.09) #	19.71 (±2.59) #	19.69 (±4.06) #

The data are presented as the means and standard deviations (in brackets). Significant differences are highlighted with * for sex at the same assessment timepoint and # for timepoints between the same sex. Differences were assessed by two-sided t tests, and significance was set at α < 0.05. avg SWV: region average shear wave velocity; CV: coefficient of variation; proximal: proximal patellar tendon region; distal: distal patellar tendon region.

### 3.3 Shear-wave velocity differences between “healthy” skiers, “symptomatic” skiers, and “healed” skiers who were symptomatic at baseline

**FIGURE 2:**
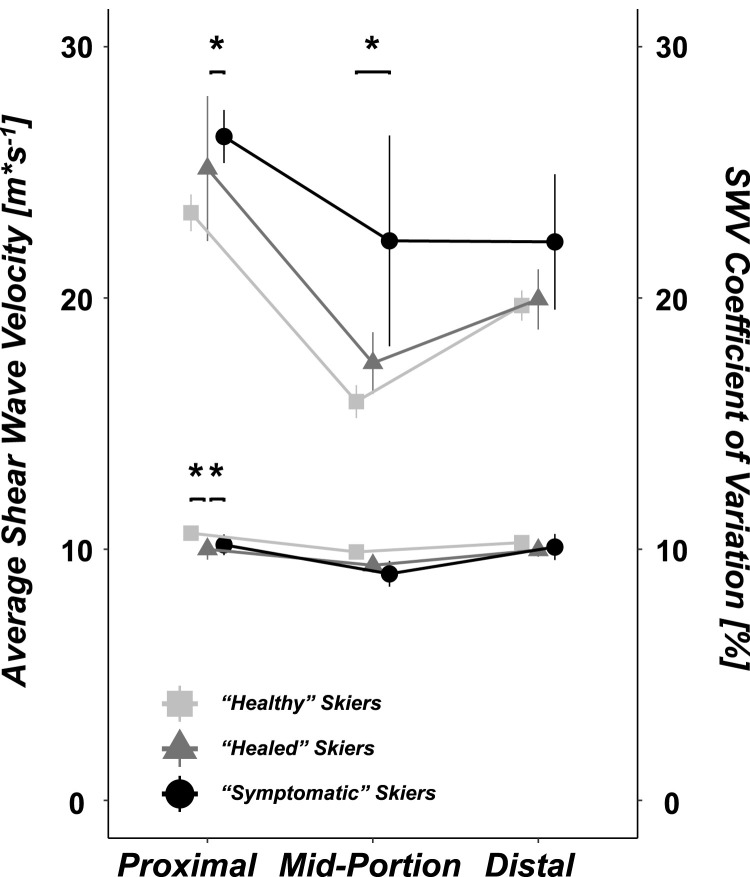
Shear wave velocity (SWV) average; light gray rectangles represent non-symptomatic skiers with no history of PT complaints (*n* = 32), dark gray triangles represent “healed” subjects who were symptomatic at baseline but healed at follow-up (*n* = 9), and black circles represent skiers who were symptomatic at follow-up (*n* = 6). The respective coefficients of variation are presented in the upper section of the figure. Significant differences are denoted by * = *p* < 0.05, and ** = *p* < 0.001.

The Kruskal-Wallis test showed that the SWV differed between “healed” skiers, “symptomatic” skiers, and “healthy” skiers in the proximal region (*p* = 0.014) but not in the mid-portion (*p* = 0.088) or distal region (*p* = 0.338). For the SWV CV, differences between the groups were detected in the proximal (*p* = 0.033) and mid-portion regions (*p* = 0.023) but not in the distal region (*p* = 0.815). The respective absolute values are presented in Table 3 and visualized in Figure 2. A more detailed data overview, separated by the values of skiers with unilateral (healthy side and affected side) and bilateral patellar tendon complaints compared to healthy skiers, can be found in the Supplementary Material.

**TABLE 3:** Shear wave velocity (SWV) average and SWV CV values of skiers who were symptomatic at baseline but who were healed at follow-up, of skiers that were symptomatic at follow-up, and of skiers with no history of patellar tendon complaints.

	Average SWV [m*s ^*-1*^ *]*			*SWV CV [%]*		
	*Proximal*	*Mid-Portion*	*Distal*	*Proximal*	*Mid-Portion*	*Distal*
*“Healed Skiers”: Symptomatic at baseline but who were healed at follow-up (n = 9)*	9.74 (±0.83) *#	9.01 (±0.66)	9.78 (±0.77)	26.95 (±2.99) #	16.70 (±2.91)	20.47 (±3.53)
*“Symptomatic Skiers”:* S*ymptomatic at follow-up (n = 6)*	10.57 (±1.37) *	9.54 (±1.40)	10.36 (±1.19)	23.72 (±10.29)	23.34 (±9.94) *°*	21.45 (±6.87)
*“Healthy Skiers”: Non-symptomatic skiers with no history of PT complaints (n = 32)*	10.64 (±0.82) #	9.89 (±1.23)	10.26 (±0.89)	23.39 (±4.14) #	15.88 (±3.72) *°*	19.70 (±3.39)

The data are presented as the mean values and standard deviations in brackets. Significant differences are highlighted with *, #, and ° for the comparisons between “Healed - Symptomatic”, “Healed–Healthy”, and “Symptomatic–Healthy”, respectively. PT: patellar tendon. Significance based on Dunn-Bonferroni tests.

The *post hoc* Dunn-Bonferroni tests revealed that in the proximal region the average SWV was lower in “healed” skiers than in both symptomatic skiers and “healthy” skiers (z = −2.437, *p* = 0.044 and z = 2.705, *p* = 0.020, respectively). The SWV CV in the proximal region was greater in “healed” skiers than in “healthy” skiers (z = −2.605, *p* = 0.028). In the mid-portion region, no significant differences in the SWV were found between the groups. Symptomatic skiers had a greater SWV CV than “healthy” skiers (z = −2.715, *p* = 0.020). In the distal region, no differences were detected between the groups for either the SWV or SWV CV.

### 3.4 The average SWV and SWV coefficient of variation are negatively correlated

**FIGURE 3:**
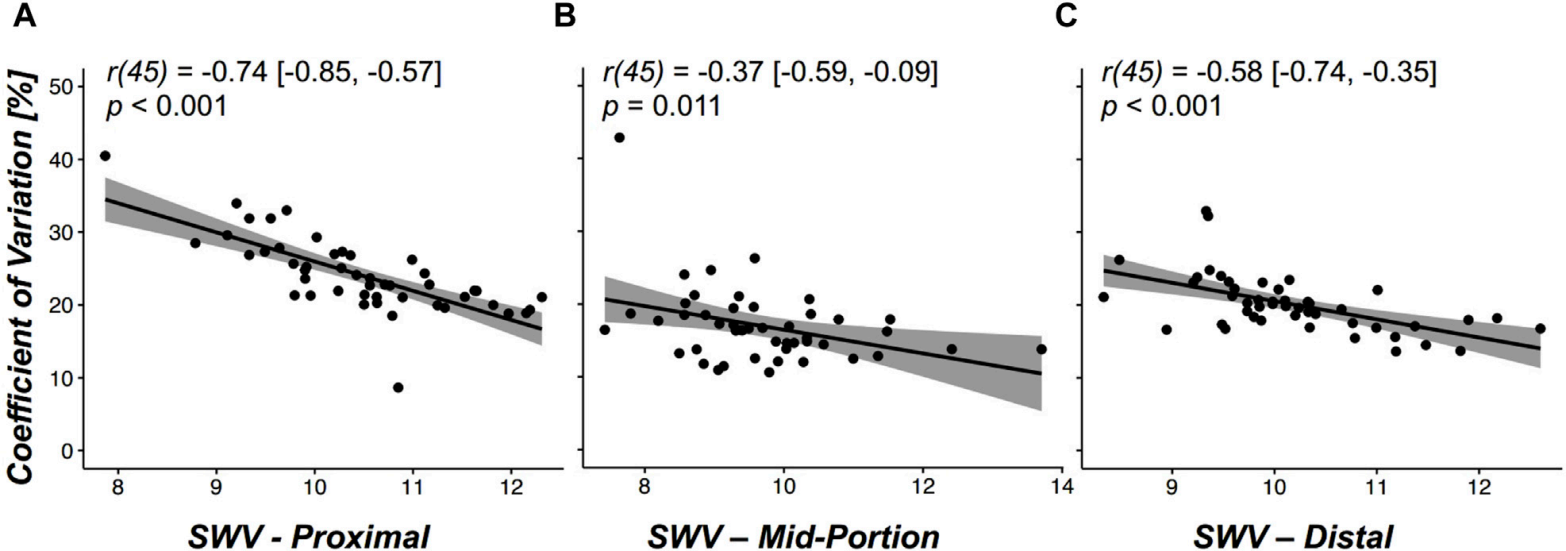
**(A–C)**: Scatter plots illustrating the correlation between the average shear wave velocity (SWV) (*x*-axis) and the coefficient of variation (*y*-axis) for assessed regions **(A)** proximal region, **(B)** mid-portion region, and **(C)** distal region and all assessed subjects (*n* = 47). Pearson correlation coefficients r (df) are presented with the lower and upper 95% confidence intervals in brackets and the corresponding *p* values.

A negative correlation between the average SWV and the SWV CV was found for the proximal (r (45) = −0.74 [−0.85, −0.57], *p* < 0.001), mid-portion (r (45) = −0.37 [−0.59, −0.09], *p* = 0.011), and distal (r (45) = −0.58 [−0.74, −0.35], *p* < 0.001) regions, as shown in Figure 3. The correlations were considered *strong* for the proximal and distal regions and *moderate* for the mid-portion region.

## 4 Discussion

This study highlights the physical and mechanical changes that PTs in adolescent skiers are subject to. The main findings of the study were as follows: 1) the prevalence of PT complaints decreased from 29.8% at baseline to 12.8% at the 4-year follow-up; 2) there were no sex differences regarding the average SWV and SWV CV in any region or at any timepoint; 3) the average PT SWV significantly decreased from the baseline assessment to the 4-year follow-up in proximal and distal regions in females and male skiers; 4) the SWV CV in the distal and mid-portion regions was significantly greater at the 4-year follow-up than at baseline; 5) at the follow-up assessment, “healed” skiers who were symptomatic at baseline but who were healed at the 4-year follow-up had an even greater average SWV drop in the proximal region and greater SWV CV in the proximal region than “healthy” skiers with no history of PT complaints, “symptomatic” skiers had higher SWV CV in the mid-portion region than “healthy” skiers; and 6) the average SWV and corresponding SWV CV showed a significant negative correlation in the proximal and distal regions.

### 4.1 The progression of PT complaint prevalence throughout adolescence

The prevalence of PT complaints significantly decreased in our cohort from baseline to the 4-year follow-up. For the interpretation of these findings, however, it must be considered that several skiers of the original cohort ended their athletic careers during the 4-year follow-up period for unknown reasons. However, the prevalence in the entire baseline cohort was comparable to that in the subcohort assessed at follow-up (31.6% and 29.8%, respectively) (Götschi et al., 2023). Thus, selection bias on account of PT complaints seems rather unlikely. Compared to that in the general population, the occurrence of PT complaints in athletes is relatively high (Nutarelli et al., 2023). Considering the drop-outs from sports, this could have contributed to the decrease in PT complaints observed in this study. In addition, a sport-specific selection bias may arise if skiers with severe PT complaints are unable to train for longer periods of time, which could ultimately affect their sports performance and end their careers (Kettunen et al., 2002; Malliaras et al., 2015; Muaidi, 2020). Moreover, Cook and colleagues reported that one-third of athletes suffering from patellar tendinopathy were not able to return to sports for more than 6 months (Cook et al., 1997). In principle, this could also explain the lower prevalence of PT complaints in the described cohort. Although the aforementioned explanation seems reasonable, it remains hypothetical since the severity of the PT complaints was not recorded. A similar interpretation may also explain the discrepancy in previous research according to which younger age has been reported as a risk factor for jumper’s knee (Zwerver et al., 2011), while common sense is that the risk for elite athletes, and hence adult athletes to suffer from PT complaints, is greater than that for youth athletes (Cook et al., 2000; Cassel et al., 2015; Florit et al., 2019; Nutarelli et al., 2023).

### 4.2 The progression of SWV patterns throughout adolescence

At the 4-year follow-up, both sexes showed a decrease in the average SWV within the proximal and distal regions. The differences were larger than the standard error of measurement reported in the proof-of-concept study (Götschi et al., 2021). However, the mid-portion did not show noteworthy differences between the measurement timepoints. This finding is in line with a study in which 12 to 14 year-old male handball players did not show PT stiffness changes over a period of approximately 10 months; however, another definition and measurement approach for PT mechanical properties was used and the participants were younger (Mersmann et al., 2021). Conversely, in another study, patellar tendon stiffness was shown to increase during adolescence; again, different assessment approaches and definitions were used (Charcharis et al., 2019). In their review, Mersmann and colleagues highlighted that muscles and tendons adapt differently to mechanical loading, and in particular, during adolescence, when maturation is an additional stimulus, the risk for muscle-tendon unit imbalances is high, potentially favouring tendinopathies (Mersmann et al., 2017).

The SWV CV in the distal and mid-portion regions was significantly greater at the 4-year follow-up than at baseline. These findings are interesting since tendons undergo different maturation-related adaptations during adolescence. Rudavsky and colleagues reported that proximal PT thickness increases during maturation, probably related to an increase in body mass and muscle strength (Rudavsky et al., 2017). Moreover, proximal tendon attachment in adolescents after their growth spurt appeared similar to that in mature subjects (Rudavsky et al., 2017). Major changes are known to occur at distal insertion sites during maturation. In particular, ossification at the tibial tuberosity, including changes within the cartilaginous attachment, is assumed to be involved in normal development (Ducher et al., 2010a; Ducher et al., 2010b). However, further investigations are needed to clarify to what degree the decrease in average SWV from baseline to the 4-year follow-up might be related to these physiological adaptations or whether this is rather an effect of excessive loading of competitive alpine skiers during adolescence.

### 4.3 The SWV average is decreased and the SWV CV is increased in skiers with a history of “healed” PT complaints

At the follow-up assessment, skiers with a history of PT complaints (i.e., skiers who were symptomatic at baseline but who were “healed” (at least 12 months symptom-free) at the 4-year follow-up) had a lower average SWV in the proximal region and greater SWV CV in the proximal region than in “healthy” subjects. These findings are in line with most of the previous literature, where a decrease in the SWV has been described in the context of PT complaints (Dirrichs et al., 2016; Dirrichs et al., 2018; Götschi et al., 2023). Conversely, there were no significant differences in the SWV between skiers who were symptomatic at the time of the follow-up assessment and “healthy” skiers, however, they had a greater SWV CV in the mid-portion region. Although variability did not differ between symptomatic and asymptomatic skiers in a previous study (Götschi et al., 2023), the present study revealed interesting effects in combination with PT complaints. A decreased SWV can be linked to known pathological processes in the context of tendinopathy that leads to decreased fibre alignment and disrupts the homogeneity of tendon tissue (Maffulli et al., 2000; Fredberg and Stengaard-Pedersen, 2008; Attia et al., 2014; Ooi et al., 2016; Snedeker and Foolen, 2017; Finnamore et al., 2019). Our findings also support the theory, that tendon repair occurs only to a certain degree but not fully (Rudavsky and Cook, 2014; Cook et al., 2016). Furthermore, our results support the hypothesis that degenerative portions of tendons are mechanically silent and not involved in remodelling (Thornton and Hart, 2011; Rudavsky and Cook, 2014; Cook et al., 2016). Interestingly, differences in the PT SWV and SWV CV were still visible in asymptomatic skiers at the follow-up. Therefore, it is plausible that skiers with a history of PT complaints have remaining structural irregularities, i.e., degeneration. Considering the follow-up period of 4 years, the reduced average SWV in skiers with a history of PT complaints may be explained by degenerative aspects within the tendons that are not involved in adaptive processes of tissue repair.

In terms of the correlation between the SWV and SWV CV, the proximal and distal regions showed a *strong* negative correlation, indicating that SWV variability is greater in tendons and regions with lower SWVs. In terms of patellar tendon complaints, these regions are certainly of high interest since they are the most frequently affected regions (Fröhlich et al., 2020; Götschi et al., 2023). The SWV CV did differ between subjects with a history of PT complaints and healthy subjects; thus, the consideration of the SWV CV in such analyses might provide valuable additional information.

### 4.4 Limitations

Despite the long follow-up period of 4 years and the use of a unique sample, this study has some limitations that should be considered when interpreting the study findings.

First, of the original pool of 108 skiers participating in the baseline assessment, only a subset of 47 could be reassessed as part of the 4-year follow-up study. Several subjects were not willing to participate in the follow-up assessment (*n* = 44), and a smaller proportion could not be included due to technical issues (*n* = 17). Second, despite good to very good inter-operator and inter-day test-retest reliability, (Götschi et al., 2021), the SWV assessments are, at least to a certain extent, operator- and day-dependent, which may limit the reproducibility of the underlying 3D SWE measurements.

Moreover, the assumption of tendon structure uniformity has been critically discussed in the literature (Ito et al., 2023). Although a regional distinction into proximal, mid-portion, and distal was made, a division along the longitudinal axis (medial, mid-portion, and lateral) was not part of this project. This extension might provide further interesting information for a comprehensive understanding of PT complaints.

## 5 Conclusion

Our 4-year follow-up study with a sample of adolescent competitive alpine skiers revealed a decrease in the prevalence of PT complaints. Moreover, at the 4-year follow-up compared to baseline, both sexes showed a decrease in the average SWV in the proximal and distal regions, as well as a greater SWV CV in the distal and mid-portion regions. Despite the decreasing prevalence of PT complaints, changes in mechanical tendon proprieties (i.e., lower average SWV and greater SWV CVs) appear to further persist in asymptomatic skiers with a history of PT complaints. Thus, long-term implications for PT properties are expected even after becoming symptom free. These observations may be indicative of degenerative processes in the patellar tendons, which are affected by complaints in maturing adolescent competitive alpine skiers, and highlight the importance of adequate load management in adolescent athletes. Interestingly, the average SWV and CV were negatively correlated, emphasizing our hypothesis that a low SWV is associated with a high CV. Accordingly, SWV variability might serve as a complementary measure in the context of patellar tendon complaint assessment using 3D SWE. However, an exact approach has not yet been defined and, thus, should be subject to further investigation.

## Data Availability

The datasets presented in this article are not readily available because their access is restricted to protect the interests of the project partner Swiss-Ski and their athletes. Requests to access the datasets should be directed to joerg.spoerri@balgrist.ch.
