# Supplementary material for: Changes in patellar tendon complaints and shear wave velocity patterns among competitive alpine skiers during a 4-year post-growth spurt follow-up
Source: Front Physiol. 2024 Jul 8;15:1401632. doi: 10.3389/fphys.2024.1401632 (PMC11261000; doi:10.3389/fphys.2024.1401632)
Supplement: Supplementary file 1 [file Table1.pdf]

Supplementary Material

Changes in patellar tendon complaints and shear wave velocity patterns among competitive alpine skiers during a 4-year post-growth spurt follow-up

Jonas Hanimann*, Daniel P. Fitze, Tobias Götschi, Stefan Fröhlich, Walter O. Frey, Eling D. de Bruin, Sutter R, Spörri J

*** Correspondence:** Corresponding Author: jonas.hanimann@balgrist.ch

1 Supplementary Tables

SKIERS WITH COMPLAINTS AT EITHER BASELINE OR FOLLOW-UP (n =15)

Table S1: *Data on the average SWV and SWV CV of healthy skiers, of skiers with unilateral patellar tendon complaints at either baseline or follow-up (n=6), and of skiers with bilateral tendon complaints at either baseline or follow-up (n=9).*

	Healthy (n = 32)	*Unilateral Complaints Healthy Side (n = 6)*	*Unilateral Complaints Affected Side (n = 6)*	*Bilateral Complaints (n = 9)*
avg SWV_proximal [m*s^{-1}]	10.64 (± 0.82)	10.68 (± 1.18)	10.60 (± 1.52)	9.73 (± 0.90)
avg SWV_mid-portion [m*s^{-1}]	9.89 (± 1.23)	9.89 (± 1.17)	9.40 (± 1.63)	9.03 (± 0.35)
avg SWV_distal [m*s^{-1}]	10.26 (± 0.89)	10.68 (± 1.25)	10.54 (± 1.46)	9.70 (± 0.38)
CV_SWV_proximal [%]	23.39 (± 4.14)	23.89 (± 4.35)	25.03 (± 3.79)	26.04 (± 8.42)
CV_SWV_mid-portion [%]	15.88 (± 3.72)	17.35 (± 5.00)	22.96 (± 10.47)	17.91 (± 3.83)
CV_SWV_distal [%]	19.70 (± 3.39)	18.86 (± 6.60)	20.45 (± 7.31)	20.89 (± 3.03)

The data are presented as the means and standard deviations in brackets. avg SWV: region average shear wave velocity; CV: coefficient of variation; proximal: proximal patellar tendon region; distal: distal patellar tendon region.

"HEALED SKIERS" – SYMPTOMATIC AT BASELINE BUT HEALED AT FOLLOW-UP (n = 9)

Table S2: *Data on the average SWV and SWV CV of healthy skiers, of "healed skiers" with unilateral patellar tendon complaints (n=3), and of "healed skiers" with bilateral tendon complaints (n=6). "Healed skiers" refers to skiers who were symptomatic at baseline but who were healed at follow-up.*

	Healthy (n = 32)	*Unilateral Complaints Healthy Side (n = 3)*	*Unilateral Complaints Affected Side (n = 3)*	*Bilateral Complaints (n = 6)*
avg SWV_proximal [m*s^{-1}]	10.64 (± 0.82)	9.90 (± 1.28)	9.78 (± 1.83)	9.69 (± 0.37)
avg SWV_mid-portion [m*s^{-1}]	9.89 (± 1.23)	9.26 (± 1.21)	8.81 (± 1.12)	9.00 (± 0.43)
avg SWV_distal [m*s^{-1}]	10.26 (± 0.89)	10.04 (± 1.60)	9.87 (± 1.13)	9.70 (± 0.42)
CV_SWV_proximal [%]	23.39 (± 4.14)	26.26 (± 5.52)	27.05 (± 2.79)	27.10 (± 2.74)
CV_SWV_mid-portion [%]	15.88 (± 3.72)	16.29 (± 4.15)	17.49 (± 4.10)	16.64 (± 3.45)
CV_SWV_distal [%]	19.70 (± 3.39)	20.45 (± 9.70)	19.93 (± 4.15)	20.65 (± 2.82)

The data are presented as the means and standard deviations in brackets. avg SWV: region average shear wave velocity; CV: coefficient of variation; proximal: proximal patellar tendon region; distal: distal patellar tendon region.

<u>"SYMPTOMATIC SKIERS" – SYMPTOMATIC AT FOLLOW-UP (n = 6)</u>

Table S3: *Data presenting the average SWV and SWV CV of healthy skiers, of "symptomatic" skiers" with unilateral patellar tendon complaints (n=3), and of "symptomatic skiers" with bilateral tendon complaints (n=3). "Symptomatic skiers" refers to skiers who were symptomatic at follow-up.*

	Healthy (n = 32)	*Unilateral Complaints Healthy Side (n = 3)*	*Unilateral Complaints Affected Side (n = 3)*	*Bilateral Complaints (n = 3)*
avg SWV_proximal [m*s^{-1}]	10.64 (± 0.82)	11.47 (± 0.02)	11.42 (± 0.65)	9.83 (± 1.70)
avg SWV_mid-portion [m*s^{-1}]	9.89 (± 1.23)	10.52 (± 0.88)	9.99 (± 2.08)	9.01 (± 0.16)
avg SWV_distal [m*s^{-1}]	10.26 (± 0.89)	11.32 (± 0.27)	11.21 (± 1.64)	9.71 (± 0.37)
CV_SWV_proximal [%]	23.39 (± 4.14)	21.53 (± 0.15)	23.02 (± 4.00)	23.94 (± 15.96)
CV_SWV_mid-portion [%]	15.88 (± 3.72)	18.41 (± 6.47)	28.43 (± 12.94)	20.46 (± 3.79)
CV_SWV_distal [%]	19.70 (± 3.39)	17.27 (± 2.68)	20.97 (± 10.75)	21.38 (± 4.03)

The data are presented as the means and standard deviations in brackets. avg SWV: region average shear wave velocity; CV: coefficient of variation; proximal: proximal patellar tendon region; distal: distal patellar tendon region.
